# Supplementary material for: Efficacy and Safety of Fecal Microbiota Transplantation in Treatment of Clostridioides difficile Infection among Pediatric Patients: A Systematic Review and Meta-Analysis
Source: Microorganisms. 2022 Dec 12;10(12):2450. doi: 10.3390/microorganisms10122450 (PMC9781859; doi:10.3390/microorganisms10122450)
Supplement: Supplementary file 1 [file microorganisms-10-02450-s001.zip › microorganisms-2056541-supplementary.pdf]

Supplemental Table S1: Search terms used to identify and filter studies from literature

Keywords	Pediatrics, *Clostridioides difficile* infection, FMT
Inclusion Criteria	• Age </= 21 yo<br>• Diagnosis of CDI, or rCDI (recurrent CDI and/or refractory CDI)<br>• FMT administered for CDI<br>• Reporting of patient data and outcomes after first fecal infusion<br>• Minimum follow-up of 8 weeks<br>• At least moderate quality of evidence - as determined by RoB 2 (Revised Cochrane risk-of-bias assessment tool) and NIH assessment or NOS (Newcastle-Ottawa Scale)<br>• At least 5 patients
Exclusion Criteria	• FMT not administered<br>• Less than 5 patients<br>• Animal studies<br>• Review articles<br>• Systematic review and meta analyses<br>• Lack of patient data
PubMed Search	Concept 1: "Fecal Microbiota Transplantation"[MeSH] OR "fecal microbiota transplantation"[tiab] OR "faecal microbiota transplantation"[tiab] OR "fecal microbiota transplant"[tiab] OR "faecal microbiota transplant"[tiab] OR "fecal microbiota transfer"[tiab] OR "faecal microbiota transfer"[tiab] OR "fecal transplant"[tiab] OR "faecal transplant"[tiab] OR "fecal transfer"[tiab] OR "faecal transfer"[tiab] OR "donor feces"[tiab] OR "donor faeces"[tiab] OR "donor stool"[tiab] OR "bacteriotherapy"[tiab] OR "FMT"[tiab]<br><br>AND<br><br>Concept 2: "Child"[Mesh] OR “child*”[tiab] OR “adolescent*”[tiab] OR “youth*”[tiab] OR “teen*”[tiab] OR “young adult”[tiab] OR "Infant"[Mesh] OR “infant*”[tiab] OR “pediatric*”[tiab]<br><br>Filter: English<br>Filter: Exclude animal studies
EMBASE Search	'fecal microbiota transplantation' AND 'pediatrics'<br><br>Age<br>Newborn (0-1 months)<br>Infant (1-12 months)<br>Child (1-12 years)

	Preschool child (1-6 years)<br>School child (7-12 years)<br>Adolescent (13-17 years)<br>Young adult (18-24 years)<br><br>Study types<br>Human<br><br>Publication type<br>Article
CINAHL Search	'fecal microbiota transplantation' AND 'pediatric'
Cochrane Search	‘Fecal microbiota transplantation’ [Abstract] AND ‘Pediatrics’ [Abstract] OR ‘Children’ [Abstract]
Google Scholar Search	"Fecal microbiota transplantation" AND "Pediatrics"

Supplemental Table S2: List of studies for which full articles were retrieved and reviewed

**Author/Year**	**Title**	**Include/ Exclude**
Nicholson 2022 [1]	Efficacy and Outcomes of Faecal Microbiota Transplantation for Recurrent *Clostridioides difficile* Infection in Children with Inflammatory Bowel Disease	Include
Li 2022 [2]	Characteristics and management of children with *Clostridiodes difficile* infection at a tertiary pediatric hospital in China	Include
Kellermayer 2022 [3]	Fecal Microbiota Transplantation Commonly Failed in Children With Co-Morbidities	Include
Aldrich 2019 [4]	Analysis of Treatment Outcomes for Recurrent *Clostridium difficile* Infections and Fecal Microbiota Transplantation in a Pediatric Hospital	Include
Nicholson 2020 [5]	Efficacy of Fecal Microbiota Transplantation for *Clostridium difficile* Infection in Children	Include
Hourigan 2019 [7]	Fecal Transplant in Children With *Clostridioides difficile* Gives Sustained Reduction in Antimicrobial Resistance and Potential Pathogen Burden	Include
Barnes 2018 [9]	Competitively Selected Donor Fecal Microbiota Transplantation: Butyrate Concentration and Diversity as Measures of Donor Quality	Include
Fareed 2018 [10]	Applying fecal microbiota transplantation (FMT) to treat recurrent *Clostridium difficile* infections (rCDI) in children	Include

Brumbaugh 2018 [26]	An Intragastric Fecal Microbiota Transplantation Program for Treatment of Recurrent *Clostridium difficile* in Children is Efficacious, Safe, and Inexpensive	Include
Hourigan 2015 [27]	Microbiome changes associated with sustained eradication of *Clostridium difficile* after single faecal microbiota transplantation in children with and without inflammatory bowel disease	Include
Barfield 2018 [25]	Going to the Bank: Fecal Microbiota Transplantation in Pediatrics	Include
Pierog 2014 [28]	Fecal microbiota transplantation via nasogastric tube for recurrent C*lostridium difficile* infection in pediatric patients	Include
Kronman 2015 [29]	Fecal Microbiota Transplantation Via Nasogastric Tube for Recurrent *Clostridium difficile* Infection in Pediatric Patients	Include
Russell 2014 [30]	Fecal transplant for recurrent *Clostridium difficile* infection in children with and without inflammatory bowel disease	Include
Zhang 2018 [43]	Safety of fecal microbiota transplantation in Chinese children: A single-center retrospective study	Exclude
Li 2018 [44]	Clinical Efficacy and Microbiome Changes Following Fecal Microbiota Transplantation in Children With Recurrent *Clostridium Difficile* Infection	Exclude
Drewes 2019 [45]	Transmission and clearance of potential procarcinogenic bacteria during fecal microbiota transplantation for recurrent *Clostridioides difficile*	Exclude
Bluestone 2018 [46]	Fecal Microbiota Transplantation for Recurrent *Clostridium difficile* Infections in Pediatric Hematopoietic Stem Cell Transplant Recipients	Exclude
Walia 2014 [47]	Efficacy of fecal microbiota transplantation in 2 children with recurrent *Clostridium difficile* infection and its impact on their growth and gut microbiome	Exclude
Ooijevaar 2019 [48]	Faecal transplants for children with recurrent infections	Exclude

Supplemental Table S3: Risk of Bias Assessment for Observational Cohort Studies with Newcastle-Ottawa Scale

	Nicholson 2022 [1]	Li 2022 [2]	Kellermayer 2022 [3]	Aldrich 2019 [4]	Nicholson 2020 [5]	Hourigan 2019 [7]	Barnes 2018 [9]	Fareed 2018 [10]	Brumbaugh 2018 [26]	Hourigan 2015 [27]
**Selection**										
1. Representativeness of the exposed cohort (Truly or somewhat)	*	*	*	*	*	no	*	*	*	*
2. Selection of the non exposed cohort (drawn from the same community as the exposed cohort)	*	*	*	*	No	No	No	No	No	No
3. Ascertainment of exposure (secure record or structured interview)	*	*	*	*	*	*	*	*	*	*
4. Demonstration that outcome of interest was not present at start of study (yes)	*	*	*	*	*	*	*	*	*	*
**Comparability**										
5. Study controls for age	*	*	*	*	*	*	*	*	*	*
6. Study controls for at least 3 additional factors	*	*	No	*	*	*	*	*	*	*
**Outcome**										
7. Assessment of outcome (independent blind assessment or record linkage)	No	No	No	*	*	*	*	*	*	*
8. Was follow-up long enough for outcomes to occur (yes)	*	*	*	*	*	*	*	*	*	*
9. Adequacy of follow up of cohorts (complete	*	*	*	*	*	*	*	*		

of small number lost)									*	*
**Total number of ***	8	8	7	9	8	7	8	8	8	8
**Score 7-9 high quality, 4-6 moderate risk of bias, 0-3 high risk of bias.**	High	High	High	High	High	High	High	High	High	High

Supplemental Table 4: Risk of Bias Assessment for Case Series with NIH quality assessment tool

**Author/Year**	**Barfield 2018 [25]**	**Pierog 2014 [28]**	**Kronman 2015 [29]**	**Russell 2014 [30]**
1. Was the study question or objective clearly stated?	yes	yes	yes	Yes
2. Was the study population clearly and fully described, including a case definition?	yes	yes	yes	Yes
3. Were the cases consecutive?	yes	no	yes	Yes
4. Were the subjects comparable?	yes	yes	yes	Yes
5. Was the intervention clearly described?	yes	yes	yes	Yes
6. Were the outcome measures clearly defined, valid, reliable, and implemented consistently across all study participants?	yes	yes	yes	No
7. Was the length of follow-up adequate?	Yes	yes	yes	Yes
8. Were the statistical methods well-described?	yes	no	No	No
9. Were the results well-described?	Yes	yes	yes	Yes
**Total quality (total number of Yes)**	9	7	8	7
**Quality (7-9 yes: Good; 4-6 yes: Fair; 1-3 yes: Poor)**	Good	good	good	Good
